# TNFα depleting therapy improves fertility and animal welfare in TNFα-driven transgenic models of polyarthritis when administered in their routine breeding

**DOI:** 10.1177/0023677217707985

**Published:** 2017-05-08

**Authors:** Amy J. Naylor, Guillaume Desanti, Atif N. Saghir, Rowan S. Hardy

**Affiliations:** 1 Institute of Inflammation and Ageing, University of Birmingham, Birmingham, UK; 2 Institute of Metabolism and Systems Research, University of Birmingham, Birmingham, UK; 3 Centre for Endocrinology Diabetes and Metabolism, Birmingham Health Partners, Edgbaston, Birmingham, UK

**Keywords:** murine polyarthritis, breeding, infliximab, refinement

## Abstract

Transgenic tumour necrosis factor alpha (TNFα)-driven models of polyarthritis such as the TNF^ΔARE^ mouse have proven to be invaluable in delineating aspects of inflammatory disease pathophysiology in humans. Unfortunately, the onset of joint destruction and inflammation in these models represents a significant detriment to breeding management. We examined whether TNFα depleting therapy ‘infliximab’ might represent a significant refinement in routine breeding. Clinical scores of joint inflammation were assessed in TNF^ΔARE^ males receiving either infliximab (10 mg/kg) or saline by twice-weekly intraperitoneal injection. Joint histology and bone morphology were assessed by histological analysis and micro-computed tomography (CT), respectively. Analysis of breeding was examined retrospectively in TNF^ΔARE^ males prior to, and following, regular introduction of infliximab. Clinical scores of inflammation were significantly reduced in TNF^ΔARE^ males receiving infliximab (control 6.6 arbitrary units [AU] ± 0.88 versus infliximab 4.4 AU ± 1.4; *P* < 0.05), while measures of pannus invasion and bone erosion by histology and micro-CT were markedly reduced. In the breeding groups, TNF^ΔARE^ males receiving infliximab injections sired more litters over their breeding lifespan (control 1.69 ± 0.22 versus infliximab 3.00 ± 0.19; *P* < 0.005). Furthermore, prior to infliximab, TNF^ΔARE^ males had a 26% risk of failing to sire any litters. This was reduced to 7% after the introduction of infliximab. This study is the first to report that regular administration of infliximab is effective at suppressing disease activity and improving animal welfare in TNF^ΔARE^ animals. In addition, we have shown that infliximab is highly efficacious in improving breeding behaviour and increasing the number of litters sired by TNF^ΔARE^ males.

## Résumé

Des modèles transgéniques TNFα de polyarthrite tels que la souris TNF^ΔARE^ se sont révélés précieux pour délimiter les aspects de la pathophysiologie des maladies inflammatoires chez les humains. Malheureusement, l’apparition de l’inflammation et la destruction des articulations chez ces modèles compromet gravement la gestion de la reproduction. Nous avons cherché à savoir si un traitement déplétif TNF a par « Infliximab » pourrait constituer une nette amélioration pour la reproduction de routine. Les résultats cliniques de l’inflammation des articulations ont été évalués chez les mâles TNF^ΔARE^ recevant soit de l’Infliximab (10 mg/kg) soit une solution saline par injection intrapéritonéale deux fois par semaine. L’histologie des articulations et la morphologie des os ont été respectivement évaluées par une analyse histologique et par micro-scanner. L’analyse de la reproduction a été réalisée rétrospectivement chez les mâles TNF^ΔARE^ avant et après l’administration régulière d’infliximab. Les résultats cliniques d’inflammation ont été réduits significativement chez les mâles TNF^DRE^ recevant de l’Infliximab (contrôle 6.6 AU + 0.88 contre infliximab, 4.4 AU ± 1.4; p < 0.05), tandis que les mesures d'invasion du pannus et de l’érosion des os par histologie et micro-scanner ont été nettement réduites. Chez les groupes reproducteurs, les mâles TNF^ΔARE^ recevant des injections d’infliximab ont engendrés plus de portées pendant leur durée de vie reproductive (contrôle 1.69 ± 0.22 contre infliximab, 3.00 ± 0.19; p < 0.005). Avant l’infliximab, les mâles TNF^ΔARE^ avaient en outre 26% de risques de ne pas pouvoir se reproduire. Ce risque a été réduit de 7% après l’administration d’Infliximab. Cette étude est la première à rapporter que l’administration régulière d’Infliximab est efficace pour supprimer l’activité de la maladie et pour améliorer le bien-être des animaux TNF^ΔARE^. De plus, nous avons montré que l’infliximab est très efficace pour améliorer le comportement reproductif et pour augmenter le nombre de portées engendrées par les mâles TNF^ΔARE^.

## Abstract (de)

Transgene TNFα-gestützte Polyarthritismodelle wie die transgene TNF^ΔARE^ Maus haben sich zur Darstellung der Pathophysiologie entzündlicher Krankheiten des Menschen als äußerst wertvoll erwiesen. Leider stellt das Auftreten von Gelenkzerstörung und -entzündung bei diesen Modellen eine erhebliche Beeinträchtigung des Zuchtmanagements dar. Wir untersuchten, ob TNFa-Blocker-Therapie eine wesentliche Verbesserung routinemäßiger Zucht bewirken kann. Klinische Gelenkentzündungswerte wurden bei TNF^ΔARE^ Männchen untersucht, denen entweder Infliximab (10 mg/kg) oder Salzlösung mittels zwei Mal wöchentlich erfolgender intraperitonealer Injektion verabreicht wurde. Gelenkhistologie und Knochenmorphologie wurden jeweils durch histologische Analyse und Mikro-CT bewertet. Die Zuchtanalyse wurde retrospektiv bei TNF^ΔARE^ Männchen vor und nach dem regelmäßigen Einsatz von Infliximab untersucht. Klinische Entzündungswerte waren bei TNF^ΔARE^ Männchen, die Infliximab erhielten, signifikant reduziert (Kontrolle 6.6 AU ± 0.88 versus Infliximab, 4.4 AU ± 1.4; p < 0.05), während mittels Histologie und Mikro-CT gewonnene Messwerte von Pannusbildung und Knochenerosion deutlich reduziert waren. In den Zuchtgruppen zeugten TNF^ΔARE^ Männchen, die Infliximab erhielten, mehr Würfe während ihrer Zuchtdauer (Kontrolle 1.69 ± 0.22 versus Infliximab, 3.00 ± 0.19; p < 0.005). Zudem bestand bei TNF^ΔARE^ Männchen vor Infliximab-Erhalt ein 26%-iges Risiko, keinen Nachwuchs zu zeugen. Nach Infliximab-Einsatz sank dieser Wert auf 7%. Mit dieser Studie wird erstmals berichtet, dass regelmäßige Infliximab-Gabe eine Hemmung der Krankheitsaktivität und eine Verbesserung des Wohlbefindens von TNF^ΔARE^ Tieren bewirkt. Außerdem wurde gezeigt, dass Infliximab höchst wirksam zur Verbesserung des Zuchtverhaltens und zur Erhöhung der Zahl der von TNF^ΔARE^ Männchen gezeugten Würfe beiträgt.

## Resumen

Los modelos transgénicos de poliartritis impulsados por TNFα como el ratón TNF^ΔARE^ han demostrado ser muy valiosos para delinear aspectos de la patofisiología de enfermedades inflamatorias en humanos. Lamentablemente, el principio de destrucción de articulaciones e inflamación en estos modelos representa un detrimento significativo para la gestión de cría. Examinamos si la terapia de agotamiento de TNFa “Infliximab” puede representar un refinamiento significativo en la cría rutinaria. Se evaluaron distintas puntuaciones clínicas de inflamación de articulaciones en machos TNF^ΔARE^ que recibían Infliximab (10 mg/kg) o salina dos veces a la semana mediante inyección intraperitoneal. La histología de las articulaciones y la morfología ósea fueron evaluadas mediante análisis histológicos y micro-CT respectivamente. Se examinaron análisis de la cría retrospectivamente en machos TNF^ΔARE^ antes y después de la introducción regular de Infliximab. Las puntuaciones clínicas de inflamación se redujeron significativamente en los machos TNF^ΔARE^ que recibían Infliximab (control 6.6 AU ± 0.88 versus Infliximab, 4.4 AU ± 1.4; p < 0.05), mientras que las medidas de invasión de pannus y erosión ósea mediante histología y micro-CT se redujeron significativamente. En los grupos de cría, los machos TNF^ΔARE^ que recibían inyecciones de Infliximab crearon más camadas en su vida útil de crianza (control 1.69 ± 0.22 versus Infliximab, 3.00 ± 0.19; p < 0.005). Asimismo, antes de Infliximab, los machos TNF^ΔARE^ tuvieron un 26% de riesgo de no poder crear ninguna camada. Esto se redujo en un 7% tras la introducción de Infliximab. Este es el primer estudio en demostrar que la administración regular de Infliximab resulta eficaz para suprimir la actividad de enfermedades y para mejorar el bienestar de animales TNF^ΔARE^. Asimismo, hemos demostrado que Infliximab es altamente eficaz para mejorar el comportamiento reproductor y para aumentar el número de camadas creadas por machos TNF^ΔARE^.

Rheumatoid arthritis (RA) is a chronic autoimmune inflammatory disease resulting in polyarticular inflammation, joint destruction and systemic inflammatory complications. Murine models of polyarthritis closely model features of RA in humans and have proven to be effective in advancing our understanding of the pathophysiology of inflammatory disease.

The primacy of murine models of inflammatory disease is underpinned by several key factors: parallels between the immune responses, their highly conserved genetic background, high progeny numbers, and short breeding times that have enabled their extensive use and validation in medical research.^1^

A range of transgenic murine models of spontaneous polyarthritis exist, including the hTNFtg, TNF^ΔARE^, KBxN, SKG and DNase II mice.^2–6^ These models offer several advantages relative to inducible models, the major benefits being the strong parallels with chronic inflammatory disease in humans and development of polyarthritis in a highly reproducible and temporally-controlled manner, making them ideal for examining therapeutic disease interventions. Indeed, in the seminal study by Keffer et al., a once-weekly injection of infliximab completely abrogated disease activity in hTNFtg mice, contributing to the now commonplace application of biological anti-tumour necrosis factor alpha (TNFα) therapies as a gold standard in the treatment of chronic inflammatory disease.^2,7,8^

The hTNFtg model (known as Tg(TNF)197Gkl) possesses an insertion of a 3′-modified human TNF transgene which greatly increases TNFα mRNA stability resulting in systemic overproduction of human TNFα.^2^ In the TNF^ΔARE^ mouse, the 3′ untranslated region (UTR) of the AU-rich element (ARE) in the murine TNFα gene is deleted, resulting in greatly increased TNFα mRNA stability and systemic overexpression.^6^ As a consequence, inflammation is driven by the overexpression of TNFα in all major tissues resulting in polyarthritis and joint destruction in both models. In addition, the TNF^ΔARE^ mouse presents with an inflammatory intestinal pathology that closely models inflammatory bowel disease as well as occasional mild inflammation in the liver and the lung. These models have helped in delineating the pathophysiology of inflammatory diseases such as RA in humans, demonstrating the role that TNFα plays at the apex of a pro-inflammatory cytokine cascade, mediating leukocyte infiltration, synovitis and joint destruction.^9^

One notable drawback associated with the use of transgenic models of polyarthritis occurs in maintaining breeding colonies required to generate experimental animals. Both hTNFtg and TNF^ΔARE^ mice are bred using a heterozygous transgenic male crossed with a wild-type female to generate offspring with a 50% incidence of developing spontaneous polyarthritis. While required in experimental animals, the spontaneous onset of polyarthritis and systemic inflammation in breeding males is of significant detriment to their welfare and breeding success.^10^

From 2–4 weeks of age, heterozygous TNF^ΔARE^ mice present with early signs of inflammatory lesions within the terminal ileum and proximal colon and become more severe around eight weeks. The histological and clinical onset of polyarthritis is detected between 6 and 8 weeks in TNF^ΔARE^ mice and becomes increasingly severe up to 14 weeks. Following onset of arthritis, animals are scored for joint inflammation, as well as behaviour, mobility, weight loss and grimace.^6^ A number of welfare measures, as outlined by Hawkins et al., are observed in the maintenance of these transgenic animals to mitigate these factors and enhance breeding. These include soft litter to reduce pain on walking, non-tangling nesting material and effortless access to food and water to cater for disability. Lastly the introduction of opiate pain relief is utilized where arthritis is evident to minimize pain and distress, given once daily at first onset (0.1 mg/kg subcutaneous buprenorphine).^11,12^ Ultimately, the introduction of clearly defined humane endpoints for articular inflammation scores (supplementary Table 1, see http://lan.sagepub.com for all supplementary material), and clinical scores of weight loss, behaviour, mobility, grimace, severity and duration of joint inflammation (supplementary Table 2), ensure that breeding males do not experience excessive distress or pain. Unfortunately, this reduces their breeding lifespan to around 14–18 weeks, relative to 35–40 weeks in wild-type animals.

Infliximab is a neutralizing and depleting antibody targeted at human TNFα, and is successfully used to ameliorate joint destruction and inflammation in hTNFtg mice.^13,14^ Interestingly, while infliximab is not reported to have cross reactivity with murine TNFα (which drives polyarthritis in the TNF^ΔARE^ model), the reported suppression of inflammation and TNFα in murine models of colitis and diabetes suggest further modes of action.^15–19^ Therefore, given the central role of TNFα in the pathophysiology of the TNF^ΔARE^ model, we examined whether a similar regimen of regular infliximab injections would improve management of our heterozygous TNF^ΔARE^ breeding programmes.

Consequently, in this study we show that regular administration of infliximab to breeding TNF^ΔARE^ males provided a significant refinement in the breeding programme, improving animal welfare by suppressing disease activity and reducing the numbers of breeding animals required to generate experimental TNF^ΔARE^ animals.

## Materials and methods

### Animals

C57BL/6 TNF^ΔARE^ mice were obtained from Dr George Kollias (BSRC Fleming, Athens, Greece).^6^ At 6–8 weeks, heterozygous TNF^ΔARE^ male mice (TNF^ΔARE/+^, later referred as TNF^ΔARE^) developed clinical signs of systemic polyarthritis, with 100% penetrance. The mice were housed under controlled environmental conditions (20.2 ± 2℃, 14:10 h light:dark cycle). The experiments were carried out following strict guidelines governed by the UK Animal (Scientific Procedures) Act 1986 and approved by the Birmingham Ethical Review Subcommittee. Only male animals were utilized for the examination of polyarthritis and breeding.

### TNF^ΔARE^ transgenic mouse model and clinical scoring

Preparations of infliximab (Remicade®; Janssen Biotech, Horsham, PA, USA) were reconstituted at 10 mg/mL in sterile water according to the manufacturer’s guidelines. To assess the effects of infliximab on disease activity, TNF^ΔARE^ males from six weeks of age were given either 10 mg/kg of infliximab or saline (100 µL of 0.9% NaCl in distilled water) by twice-weekly intraperitoneal injection. For scoring joint inflammation and polyarthritis, infliximab injections were split to ensure that within any one litter, one male TNF^ΔARE^ sibling would receive infliximab and one would receive the saline control. Animals were then scored separately in a blinded manner. TNF^ΔARE^ animals were caught by their tail, lifted with hand support and immediately transferred onto a metallic cage to minimize involuntary extension/flexion of arthritic joints. They were then restrained by the back and lifted to perform paw monitoring. The animals were familiarized with handling and observation for one week prior to the onset of experiments to minimize stress. All injections were performed on Tuesdays and Fridays in the afternoon by a trained member of staff. Upon starting infliximab the mice were scored three times weekly (Monday, Wednesday and Friday) for inflammatory joint scores (supplementary Table 1) and collective clinical scoring of weight loss, behaviour, mobility, grimace, severity and duration of joint inflammation (supplementary Table 2) as previously reported.^20^

At 14 weeks old the animals were culled and their hindlimbs were collected for histology and micro-computed tomography (CT) analysis.

### TNF^ΔARE^ transgenic mouse breeding programme

A retrospective analysis of breeding was performed in 17 breeding pairs prior to the introduction of infliximab and in 14 breeding pairs following the regular introduction of infliximab. Breeding pairs consisted of a C57BL/6 TNF^ΔARE^ male and a C57BL/6 wild-type female paired at six weeks of age. In each breeding pair, the number of successfully weaned litters during their breeding lifespan, average litter size, numbers of male and female pups per litter, litters that failed to progress through weaning, and breeding pairs that failed to produce a litter were recorded. Breeding outputs were compared with a C57BL/6 wild-type colony consisting of 10 breeding pairs, monitored between six and 26 weeks, at a time point matched to TNF^ΔARE^ animals prior to the introduction of infliximab.

### Micro-CT

The hindlimbs were isolated post-mortem and imaged with a SkyScan 1172 micro-CT scanner (Bruker, Kontich, Belgium) using X-ray beam settings of 60 kV source voltage and 167 μA source current. Projections were taken every 0.45° at 600 ms exposure, with an image pixel size of 13.59 µm. Image volumes were reconstructed using the Feldkamp algorithm (NRecon 1.6.1.5; Bruker), having applied beam hardening correction. A radiodensity range of –300 to 3000 HU was chosen to isolate the bony structures from the imaging medium. CTAnalyser 1.12 software (SkyScan) was used to extract an isosurface mesh representation of the reconstructed micro-CT slices. MeshLab 1.3.2 (an open source software developed with the support of the 3D-CoForm project) was used to modify the raw meshes, and the samples were shaded in MeshLab using ambient occlusion.

Meshes were visualized in MeshLab and scored blind by three independent researchers as follows. Meshes were divided into three regions, ‘heel’ (comprising the calcaneus, centrale, distal tarsals, tibiale and talus but excluding the tibia and fibula), ‘metatarsals’ and ‘phalanges’ (excluding the claws). Each region was scored separately for the degree of erosion: 0 = normal, 1 = roughness, 2 = pitting, 3 = full thickness holes. A score was also given to describe the extent of the area affected: 0 = none, 1 = a few small areas, 2 = multiple small–medium sized areas, 3 = multiple medium–large areas or extensive. The two scores were then multiplied together for each region, and then summed to give a maximum score per paw of 27.

### Histological analysis

Following decalcification in 0.5 M EDTA (pH 8.0), histological analysis was performed on paraffin-embedded 10 µm sections of hindlimbs taken from either wild-type or TNF^ΔARE^ animals receiving infliximab or saline injections. The sections were stained with haematoxylin and eosin prior to quantitative analysis of the pannus size at the metatarsal–phalangeal joint interface using ImageJ software (NIH, Bethesda, MD, USA). Pannus size in arbitrary units (AU) was calculated from the region of synovial pannus, clearly visible by histology (as depicted within supplementary Figure 1), and invading into the subchondral bone of the first proximal phalanges at the metatarsal–phalangeal joint interface. Three adjacent 10 µm sections were cut from the centre of the joint, and the pannus size was determined as described above to generate a mean value.

### Statistical analysis

To detect a 40% decrease in joint inflammation scores four experimental animals were required per group (calculated using in-house preliminary data in TNF^ΔARE^ mice at week 14; score 6.6, standard deviation [SD] 1.7, power 0.8, alpha 0.05). Unless stated otherwise, data shown are mean ± standard error (SE) of the mean of four wild-type control animals or four TNF^ΔARE^ animals receiving saline and five animals receiving infliximab. Statistical significance was defined as *P* < 0.05 (*P < 0.05; **P < 0.005; ***P < 0.0005) using either an unpaired Student’s *t*-test or a two-way analysis of variance (ANOVA) with a Tukey post hoc analysis where a Gaussian distribution was identified or determined using a non-parametric Kruskal–Wallis test with a Dunn’s multiple comparison where it was absent.

## Results

### Infliximab suppresses disease activity in TNF^ΔARE^ mice

**Figure 1.**
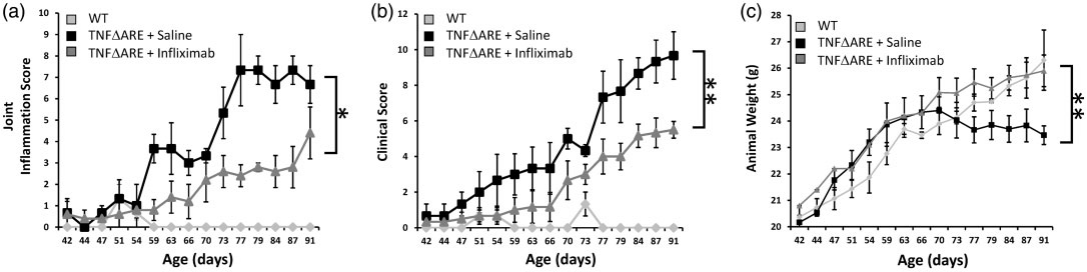
Scoring of (a) joint inflammation, (b) clinical scoring (weight, inflammation, grimace, behaviour, mobility, inflammation severity and duration), (c) total body weights in wild-type (WT) or TNF^ΔARE^ animals receiving either twice-weekly intraperitoneal injections of saline or infliximab at 10 mg/kg. Values are expressed as mean ± standard error of four WT animals, five TNF^ΔARE^ animals receiving infliximab and four TNF^ΔARE^ animals receiving saline. Statistical significance was determined using one-way analysis of variance (ANOVA) with a Tukey’s post hoc analysis. *P < 0.05, **P < 0.005.

Clinical signs of joint inflammation were evident in both saline- and infliximab-treated mice from six weeks of age, but were significantly reduced in the group receiving infliximab at eight weeks of age (Figure 1a). Joint inflammation scores remained significantly lower in the infliximab group relative to the saline group up to 14 weeks. When combined clinical scores measuring severity of inflammation, duration of inflammation, mobility, behaviour, weight and grimace were assessed, a similar pattern was observed, with increased scores being evident in saline-treated animals from eight weeks (Figure 1b). Once again, this pattern continued up to 14 weeks.

In this study, progressive weight loss provides an effective readout of the degree of systemic inflammation. Consequently, weight loss of greater than 20% also represents an effective humane endpoint in TNF^ΔARE^ mice. TNF^ΔARE^ animals receiving saline only, developed evidence of weight loss at day 70, plateauing from day 77. This was in contrast to progressive weight gain observed throughout the infliximab-treated TNF^ΔARE^ animals and wild-type counterparts (Figure 1c). Consequently, TNF^ΔARE^ animals receiving infliximab maintained a normal body weight relative to control animals throughout the monitoring period (Figure 1c).

Together, these data demonstrate that TNF^ΔARE^ animals receiving regular injections of infliximab, while not completely protected from the detrimental effects of TNFα excess, present with a markedly milder phenotype than untreated counterparts. This is evidenced by their significant reduction in clinical scores of joint inflammation, improved mobility, behaviour and grimace and improved body weights relative to TNF^ΔARE^ animals receiving saline only. Therefore, the administration of infliximab represents a significant refinement in regard to improving animal pain and welfare in the long-term maintenance of these animals.

### Infliximab protects against joint destruction

While scoring of inflammation and clinical features of disease provide valuable insight into observable measures of disease activity, they are limited in their ability to assess quantifiable measures of direct joint destruction. To gain a more comprehensive understanding of how administration of infliximab directly influences the process of joint destruction we examined three-dimensional reconstructions of joints using micro-CT, and assessed joint histology of TNF^ΔARE^ animals receiving either saline or infliximab.

**Figure 2.**
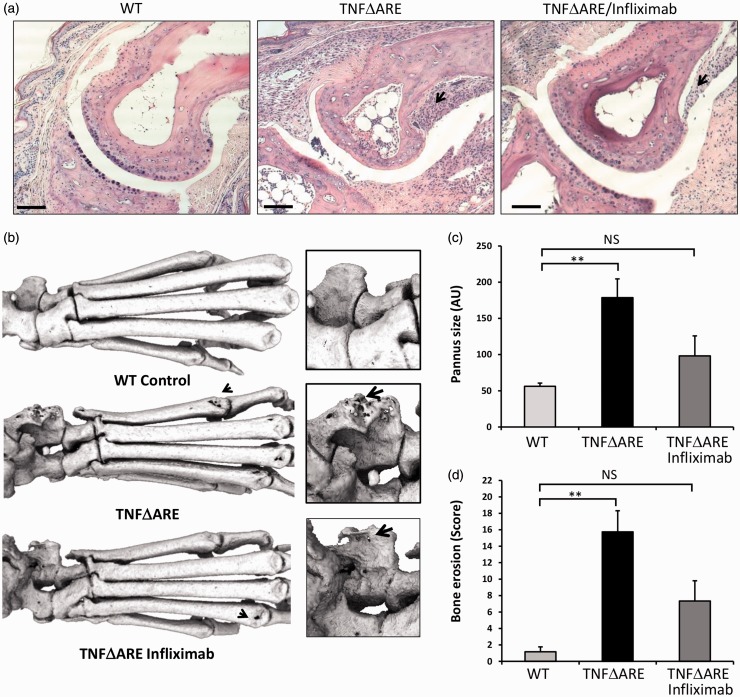
(a) Representative paraffin-embedded sections from the metatarsal–phalangeal joint interface stained with haematoxylin and eosin (scale bars 200 µm). (b) Representative images of 3D reconstructions of hind paws with increased magnification at the ankle, using micro-computed tomography(CT). (c) Quantification of degree of pannus invasion in the metatarsal–phalangeal joint interface (from haematoxylin and eosin histology). (d) Quantification of cortical erosion in the bones of the ankle, metatarsals and phalanges from micro-CT in wild-type (WT) or TNF^ΔARE^ animals receiving either twice-weekly intraperitoneal injections of saline or infliximab at 10 mg/kg. Values are expressed as mean ± standard error of four WT animals, five TNF^ΔARE^ animals receiving infliximab and four TNF^ΔARE^ animals receiving saline. Statistical significance was determined using one-way analysis of variance (ANOVA) with a Tukey’s post hoc analysis for pannus invasion and Kruskal–Wallis test and Dunn’s multiple comparison for micro-CT quantification of erosions. Arrows denote pannus adjacent to cortical bone in (a), and full thickness cortical bone erosions within (b). **P < 0.005, NS: not significant.

Analysis of cortical bone erosion by micro-CT within ex vivo hind paws of TNF^ΔARE^ animals receiving saline only, revealed significant full thickness holes in the talus, intermedium and centrale with marked pitting and roughness of the distal tarsals relative to their wild-type counterparts. By contrast full thickness cortical holes and pitting were significantly reduced in TNF^ΔARE^ animals receiving infliximab (Figure 2b). Quantification of erosions revealed a strong trend towards reduced scores in TNF^ΔARE^ animals receiving infliximab relative to those receiving saline which, unlike TNF^ΔARE^ animals, were not significantly greater than wild-type controls (Figure 2d).

The mice receiving regular infliximab injection displayed a marked reduction in inflammatory pannus in the metatarsal–phalangeal joint interface (Figure 2a). When the pannus invading within the subchondral bone was quantified, pannus size was found to be significantly reduced in TNF^ΔARE^ mice receiving infliximab relative to those receiving saline (Figure 2c).

These data demonstrate that TNF^ΔARE^ animals receiving infliximab have a significant reduction in joint inflammation and destruction relative to those receiving saline only. Similarly, local bone erosions and destruction of juxta articular bone within inflamed joints are also significantly reduced in TNF^ΔARE^ animals receiving infliximab. These data directly support clinical scoring of joint inflammation and confirm that infliximab significantly retards the disease process that underpins deformity, loss of function and joint pain.

### Infliximab increases litter numbers and breeding behaviour in TNF^ΔARE^ mice

To examine the impact of the introduction of infliximab on breeding behaviour and breeding output, we retrospectively examined 17 TNF^ΔARE^ breeding pairs from a time point prior to the introduction of infliximab injections, and 14 TNF^ΔARE^ breeding pairs following the introduction of infliximab injections.

The findings (Table 1) clearly demonstrated that TNF^ΔARE^ males receiving regular infliximab injections sired more litters over their breeding lifespan. Furthermore, when compared with wild-type control breeding pairs on a C57BL/6 background (Table 2), there was a significant decrease in the percentage of TNF^ΔARE^ males successfully siring a litter. By contrast, no significant differences were identified in TNF^ΔARE^ males successfully siring a litter following the introduction of infliximab. Prior to the introduction of infliximab injections no TNF^ΔARE^ males reached the 26-week breeding lifespan due to humane endpoints being exceeded. By contrast, following the introduction of infliximab all except three breeding males achieved a 26-week breeding lifespan (data not shown).

**Table 1.** Mean (AVG) number of litters successfully weaned, mean number of pups generated per litter, mean number of male and female pups per litter, percentage of litters that successfully survive beyond weaning and percentage of males that successfully sire a litter in breeding pairs consisting of heterozygous TNF^ΔARE^ males and wild-type (WT) females over periods preceding the introduction of regular infliximab injections, or following the regular introduction of twice weekly intraperitoneal injections of infliximab at 10 mg/kg in breeding males.

TNF^ΔARE^ male × WT female	Pre infliximab		Post infliximab		
	AVG	SE	AVG	SE	Significance
Litters weaned	1.69	0.22	3.00	0.19	0.0019
Total pups per litter	5.66	0.47	6.69	0.48	0.085
Males per litter	2.61	0.32	3.45	0.39	0.086
Female per litter	3.05	0.38	3.24	0.29	0.635
Litters successfully weaned (%)	74.13	8.65	93.46	1.28	0.0604
Successfully sire a litter (%)	70.60	14.87	85.72	9.70	0.282

Values are expressed as mean ± standard error (SE) from 17 breeding pairs where male TNF^ΔARE^ animals did not receive infliximab injections versus 14 breeding pairs where male TNF^ΔARE^ animals received regular infliximab injections. Statistical significance was determined using an unpaired student’s *t*-test for litter size and pups for litter and a Mann–Whitney *U*-test for percentage of litters weaned and percentage successfully siring a litter.

**Table 2.** Mean (AVG) number of litters successfully weaned, mean number of pups generated per litter, mean number of males and female pups per litter, percentage of litters that successfully survive beyond weaning and percentage of males that successfully sire a litter in breeding pairs consisting of wild-type (WT) males and WT females over the periods preceding the introduction of regular infliximab injections.

WT male × WT female	WT breeding		Versus TNF^ΔARE^	Versus TNF^ΔARE^/ infliximab
	AVG	SE	Significance	Significance
Litters weaned	3.80	0.39	0.0016	0.065
Total pups per litter	6.43	0.80	0.27	0.63
Males per litter	3.41	0.53	0.10	0.93
Female per litter	3.02	0.39	0.95	0.54
Litters successfully weaned (%)	86.77	10.16	0.883	0.178
Successfully sire a litter (%)	100	0.00	0.136	0.146

Values are expressed as mean ± standard error (SE) from 10 breeding pairs. Statistical significance was determined using an unpaired student’s *t*-test for litter size and pups for litter and a Mann–Whitney *U*-test for percentage of litters weaned and percentage successfully siring a litter.

These data demonstrate clear benefits of regular administration of infliximab in breeding TNF^ΔARE^ animals, resulting in greater numbers of litters over their breeding lifespan, a greater chance of siring a litter, and trends towards larger litter sizes and greater chances of litters surviving past weaning. Consequently, fewer breeding pairs are required to generate experimental animals, allowing a significant reduction in TNF^ΔARE^ breeding animals needed to maintain a breeding programme.

## Discussion

Transgenic, spontaneous mouse polyarthritis models are widely used within the rheumatology research community. Unfortunately, one significant drawback in their use in medical research arises through reduced animal welfare as a result of systemic inflammation in breeding animals used to generate experimental animals. While the 100% penetrance of disease symptoms in animals carrying a disease transgene is a benefit in helping to reduce experimental animal numbers, it is a detriment in breeding programmes where it suppresses breeding behaviour, increases pain and distress and ultimately reduces breeding lifespans of animals.

In this study we have explored whether the biological anti-human TNFα drug infliximab may prove efficacious in improving animal welfare and breeding behaviour in male TNF^ΔARE^ mice. Infliximab has previously been shown to be highly efficacious in abrogating disease activity and joint destruction in the hTNFtg mouse when given once weekly at 8 mg/kg by intraperitoneal injection, and is routinely administered in our breeding programmes of these animals.^2,20^

While hTNFtg and TNF^ΔARE^ models of polyarthritis are driven by a common pathophysiology, infliximab is not reported to cross react with murine TNFα.^21^ However, its efficacy has been reported in several murine models of chronic inflammation suggesting further modes of action beyond direct neutralization and depletion of TNFα.^15–19^ These include the induction of T-cell apoptosis and suppression of angiogenesis, possibly mediated via Fc-mediated interactions and the natural immunogenicity of infliximab.^21–24^

Therefore, we predicted that infliximab may prove effective in suppressing disease activity in TNF^ΔARE^ animals in an identical manner as reported in hTNFtg mice.^25^ Previous studies using infliximab in mice have demonstrated that it is well tolerated at levels of up to 10 mg/kg over a period of up to 24 weeks.^26^ Consequently, following consultation with the named animal care and welfare officer and named veterinary officer within our animal handling facility, we examined whether twice-weekly 10 mg/kg intraperitoneal injections of infliximab would improve animal welfare and breeding in TNF^ΔARE^ animals.

We found that clinical scoring of joint inflammation, duration of inflammation, mobility, behaviour and body weight were all significantly improved in TNF^ΔARE^ animals receiving infliximab relative to those receiving saline alone. Unfortunately, as opposed to administration of infliximab in hTNFtg animals, clinical signs of polyarthritis were still evident, indicating that regular infliximab injections were unable to completely abrogate host murine TNFα overexpression. Infliximab was originally designed to target and deplete human TNFα, and therefore may utilize alternative modes of action in suppressing disease activity in inflammatory models driven by murine TNFα. Therefore, it is unsurprising that infliximab injections are less effective at suppressing disease in this model. Despite this, it is clear that regular administration of infliximab significantly suppresses polyarthritis disease activity and improves animal welfare. This was more evident when the structure of bone by micro-CT and the histology of joints were examined, showing significantly reduced pannus invasion and bone erosions in affected joints.

While disease activity was not completely suppressed in TNF^ΔARE^ animals receiving infliximab, body weights were markedly increased relative both to those receiving saline only and to their wild-type counterparts. Weight loss in TNF^ΔARE^ animals can in part be attributed to malnutrition secondary to intestinal inflammation. Whether the weight gain observed in the infliximab-treated group reflects the suppression of inflammatory bowel disease-like symptoms cannot be determined from this study without examining intestinal pathology. This is further complicated, as rapid weight loss is also an important measure of deteriorating health in cytokine-driven cachexia. TNFα directly induces numerous catabolic pathways in mice and humans, and also suppresses normal feeding behaviour.^27–29^ Regardless of the underlying mechanism, when combined with our observations with disease activity and joint destruction, this study clearly demonstrates that regular administration of infliximab in TNF^ΔARE^ animals represents a significant refinement in their maintenance in breeding programmes, and markedly improves animal welfare.

Retrospective examination of the impact of administration of infliximab in TNF^ΔARE^ breeding males demonstrated that it was highly efficacious in improving breeding behaviour, increasing the number of litters sired during their breeding lifespan and increasing the percentage of males that successfully bred and sired litters. In addition, non-significant trends were observed in the numbers of pups sired per litter, favouring increased numbers of males per litter. This result was perhaps unsurprising given that these mice survive longer without exceeding the criteria for their humane endpoints; and it also undoubtedly reflects an improvement in animal welfare through reduced pain and distress – factors that are known to suppress mating behaviour in male rodents.^10^

Taken together, these results clearly demonstrate that the application of regular infliximab injections in TNF^ΔARE^ breeding males represents a significant refinement in practice, allowing a reduction in animal numbers required in routine breeding to generate experimental animals.

A final consideration should be whether regular infliximab administration would prove beneficial in other genetically manipulated models of spontaneous polyarthritis, such as the KBxN, SKG and DNase II mice. These models have varying involvement for TNFα in their disease pathology, and so the beneficial effects may vary greatly. Consequently, we propose that its application in these models might merit investigation.

## Conclusions

This study is the first to report that regular administration of infliximab is effective at suppressing disease activity systemic inflammation and improving animal welfare in TNF^ΔARE^ animals. In addition, we have shown that infliximab is highly efficacious in improving breeding behaviour and increasing the number of litters sired by TNF^ΔARE^ males. Consequently, in both hTNFtg and TNF^ΔARE^ mice, regular administration of infliximab in breeding males represents a refinement in routine maintenance and allows a significant reduction of the numbers of breeding animals required to generate experimental animals.

## Supplementary Material

Supplementary material
