# Supplementary material for: TNFα depleting therapy improves fertility and animal welfare in TNFα-driven transgenic models of polyarthritis when administered in their routine breeding
Source: Lab Anim. 2017 May 8;52(1):59–68. doi: 10.1177/0023677217707985 (PMC5802519; doi:10.1177/0023677217707985)

Score	Signs
1	Normal
2	Erythema and mild swelling confined to the hock joint
3	Erythema and mild swelling extending from the hock or carpus to the metatarsal or metacarpal joints
4	Erythema and moderate swelling extending from the hock or carpus to metatarsophalangeal or metacarpalophalangeal joints

Supplementary Table 1: Inflammatory joint score. From 5 weeks animals were scored for joint inflammation three times a week. Mice were scored using a 16 point system. A single point is awarded for inflammation of any one digit with the maximum score for each limb being 4 as outlined in supplementary table 1. Mice are scored using a 16 point system. An inflammatory joint score of >7 is an indication to initiate pain relief. A score of >12 is classed as a humane endpoint.

Behaviour (assessed in home cage prior to handling)	
Normal interactions with cage mates	0
Reduced interest in roaming behaviour	2
Isolated from cagemates	5
**Mobility (assessed in a separate cage)**	
Normal	0
Abnormal gait	1
Paddling	2
Reluctance to stand up on hind legs	3
Absence of load bearing	5
**Body weight**	
Normal (within 10% of age matched control)	0
>10 % weight loss	2
>15% weight loss	5
**Mouse Grimace Scale**	
Not present	0
Mild	1
Moderate	2
**Arthritic paw score (calculated from inflammatory score in table 1)**	
Normal	0
Total 1 - 3	1
Total 4 – 7	2
Total 8– 10	3
Total 11 - 12	4
**Time since first signs of arthritis were detectable**	
0-1 week	0
1-2 weeks	1
2-4 weeks	2
>4 weeks	4
**Total score**	
Sum all parameters	/25

Supplementary Table 2: Clinical scoring criteria for determination of systemic inflammatory features. From 5 weeks, mice were scored three times weekly. Clinical scores were calculated as the cumulative values from scoring behaviour, mobility, weight loss, mouse grimace, evidence of joint inflammation (supplementary Table. 1) and duration of joint swelling. A combined clinical score of >6, evidence of paddling, or isolation from cage mates

is an indication to initiate pain relief. A score of >12, loss of >20% body weight, or absence of load bearing is classed as a humane endpoint.

Supplementary figure 1: Quantification of synovial pannus invading into subchondral bone using Image J software. Pannus size in arbitrary units (AU) was calculated from the region of synovial pannus outlined in yellow, invading into subchondral bone of the first proximal phalanges at the metatarsal-phalangeal joint interface. a-b, wild type control joints, c-d, TNFDARE joints. Three adjacent 10 μm sections were cut from the centre of the joint, and pannus size determined as described above to generate a mean value.

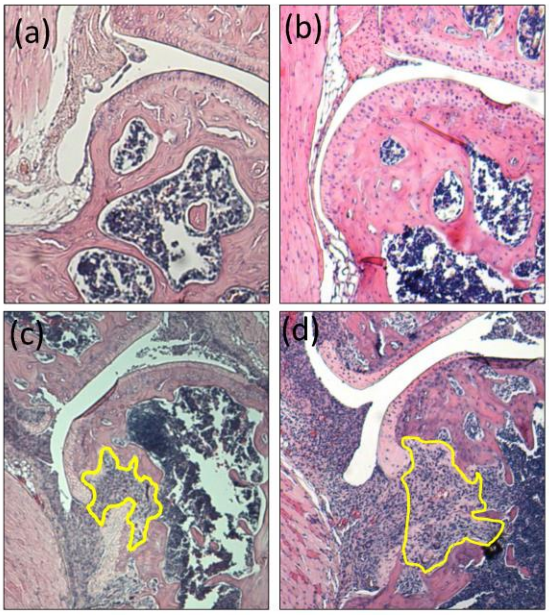

Supplement: Supplementary material [file LAN707985_supplementary_material.pdf]
